# Mental Health and Rheumatoid Arthritis: Toward Understanding the Emotional Status of People with Chronic Disease

**DOI:** 10.1155/2019/1473925

**Published:** 2019-02-11

**Authors:** Michał Ziarko, Katarzyna Siemiątkowska, Michał Sieński, Włodzimierz Samborski, Joanna Samborska, Ewa Mojs

**Affiliations:** ^1^Institute of Psychology, Adam Mickiewicz University in Poznan, Poland; ^2^Department and Clinic of Rheumatology and Rehabilitation, Poznan University of Medical Sciences, Poland; ^3^Department of Stomatological Surgery and Periodontology, Poznan University of Medical Sciences, Poland; ^4^Department of Clinical Psychology, Poznan University of Medical Sciences, Poland

## Abstract

**Introduction:**

Rheumatoid arthritis (RA) is a long-term disorder significantly impairing the somatic, emotional, and psychological functioning of its sufferers. Previous research has shown that affected individuals are characterized by an increased level of anxiety and depression. Currently, there are two main treatment schemes for RA; the first uses anti-inflammatory drugs, and the second utilizes biologic agents. This begs the question whether sufferers differ in intensities of pain, anxiety, and depression depending on the type of treatment and what the determinants of these affective states in patients treated using different methods are.

**Methods:**

The study comprised 85 patients affected by RA (including 57 receiving biologically inactive medication). Research participants filled out a set of questionnaires measuring levels of anxiety and depression, intensity of experienced pain, strategies of coping with pain, and ego resiliency.

**Results:**

The collected data was analyzed through intergroup comparisons, calculating simple correlation coefficients, developing and solving regression equations. The results imply that the choice of treatment differentiates the intensity of pain experienced by patients. Those receiving biologic agents reported lower levels of pain compared to those taking anti-inflammatory medication. It has also been noted that there are distinct configurations of conditions conducive to anxiety and depression in both anti-inflammatory and biologic agent groups.

**Discussion:**

The observed constellation of dependencies between variables indicates that the choice of treatment scheme differentiates pain levels. This confirms the assumption that pain intensity, coping strategies, and ego resiliency depend on the severity of anxiety and depression.

## 1. Introduction

Chronic illness is indicated by the World Health Organization as the leading cause of premature death in the world. According to WHO's estimates, it is responsible for 63% of all fatalities [1]. Chronic illness is defined by its slow progression and long duration, two traits which force patients to adapt to new, changed circumstances, and which affect most aspects of life, usually negatively, consequently significantly lowering health-related quality of life [2].

One chronic illness severely altering its sufferers' ability to function is rheumatoid arthritis (RA). RA is the most common rheumatic disorder among connective tissue disorders. It is a persistent, progressive inflammatory process beginning in the synovial membrane, leading to the deformation and destruction of articular tissues, and the impairment of articulatory function [3]. Typical age of onset is between 40 and 60 years and incidence is 3 to 4 times higher in women than in men. A person affected by rheumatoid arthritis experiences numerous somatic problems, such as the deformation and deterioration of joints, chronic pain, fatigue, weight loss, and fever. Besides these, the sufferer must also deal with psychological hardships, primarily marked by negative affect: anxiety, depression, feelings of loss, and social difficulties related to changes in fulfilling social roles [4].

The theoretical approach based on which we can understand the processes of adaptation to chronic disease is the Transactional Model of Stress and Coping [5]. This approach assumes that a stress transaction is a complex process in which a number of consecutive phases can be distinguished: the occurrence of an event, its cognitive evaluation, dealing with its consequences. Additionally, the stress transaction process is modified by the available resources [6]. In this perspective, resources act as a mediator between the different stages of a stress transaction. For example, due to its high mental resilience, a person is able to flexibly adjust coping strategies to the requirements of the situation [7]. In the proposed study, we investigated coping (coping with pain), resources (ego-resilience), and consequences (pain, depression, and anxiety).

A basic problem that RA patients must cope with is pain. As the disorder advances, pain levels usually increase [8]. The unpredictability of pain is one trait disrupting well-being; patients cannot predict the end of an ongoing episode of pain nor the onset of another one. This negatively impacts the sufferers' emotional state and greatly increases their negative affect.

Among the psychological consequences of RA, in the foreground are changes in the sufferer's emotional life, considered an effect of pain and growing impairment [9]. Individuals affected by RA experience anxiety and depressive symptoms to a greater degree than the general population. It is estimated that between 14% and 62% of those afflicted with RA also suffer from depression [10–14]. Its occurrence is explained through either neuroimmunobiological or psychological mechanisms. The neuroimmunobiological hypothesis points to proinflammatory cytokines, responsible for disrupting the serotoninergic system, as playing the dominant role in the development of depressive symptoms [15]. The psychological approach, on the other hand, assumes that increasing impairment resulting from gradual deterioration of joint function causes feelings of helplessness, powerlessness, and worthlessness, which contribute to the emergence and persistence of depressive symptoms.

Research on the consequences of depression in RA patients indicates that individuals exhibiting complex depressive symptoms are more susceptible to repeated recurrence of intense pain [16–18]. Experiencing depressive symptoms further hinders coping and living with illness, which manifests as frequent hospitalizations and medical appointments among other things [19]. The question remains open whether a lower mood and exhibiting symptoms of depression are antecedents sensitizing to pain, or consequences of pain [20]. Research results are inconclusive. A two-way dependency is described: on one side, the level of depression makes it possible to predict the degree of future physical and psychological impairment; on the other, physical limits caused by illness are predictors of future depression levels [21]. As such, the dependencies have the properties of a vicious cycle.

Another emotional state typical for RA is anxiety. The proportion of individuals with an increased level of anxiety stands between 21% and 70% of sufferers [13]. Some researchers suggest that symptoms of anxiety surpass depression levels in this group [22, 23]. Frequently, the sufferer fears a relapse of illness accompanied by intense pain. In many cases, this fear escalates into panic. Since fear and anxiety function as motivators to avoid threatening circumstances [24], this may lead sufferers to avoid any situations where pain could be exacerbated, especially those connected with professional or everyday activity [25].

Using the transactional model of stress [5, 26], potential determinants of anxiety and depression are the intensity of pain (specific element of the stressful life event), strategies for coping with pain, and ego resiliency. RA sufferers face the necessity of managing many hardships and problems caused by the disorder, the most important including experiencing pain, limited mobility, and uncertainty as to the disorder's further development. Among these challenges, pain probably disrupts the sufferer's everyday life to the highest degree; hence one of the most commonly posed research questions regarding this group is how they cope with pain and how their coping strategies influence their functioning. Therefore, it becomes essential to isolate specific coping strategies, which provide affected individuals with the greatest relief from pain [27].

One classification of strategies of coping with pain was proposed by Rosenstiel and Keefe [28]. They distinguished seven strategies: diverting attention, reinterpreting pain sensations, catastrophizing, ignoring pain sensations, praying and hoping, coping self-statements, and increasing activity levels. The strategies are assigned to three factors: cognitive coping and suppression, diverting attention and substitute activities, and catastrophizing.

Ego resiliency is another potentially significant condition. It is the individual's ability to adapt their level of control over internal impulses to the requirements of the current situation [29]. Ego resiliency is considered to be one of the most important positive strategies for adapting to stressful situations, as it is assigned the role of managing coping strategy choice [11, 30]. In the context of illness, individuals with a high level of ego resiliency use more effective (compared to low-resiliency individuals) crisis-coping techniques; they are therefore more capable of balancing the positive and negative emotions that they experience and exhibit a higher overall degree of self-control. A relationship between ego saliency and a tendency to choose strategies involving active coping has also been noted, which translates into increased efficacy and feelings of agency.

The pathogenesis of rheumatoid arthritis is not fully identified. RA is treated as an autoimmune disorder, wherein white blood cells attack the organism's own tissues. This process manifests in the presence of immune complexes in the synovial fluid, synthesized with the involvement of the rheumatoid factor (RF), that is, antibodies against IgG. It is assumed that the rheumatoid factor initiates and maintains the inflammatory process in joints, though how and why it starts and sustains the course of illness remains an unknown. One popular hypothesis is that of viruses or bacteria triggering [31, 32].

Because the primary cause of rheumatoid arthritis has not been discovered, treatment is symptomatic. The goal of pharmacological therapy is to halt the progress of disease and so induce remission. The drugs used over the course of RA largely have analgesic, anti-inflammatory, or immunosuppressive properties. They can modify the course of the underlying condition (most desirable) or merely ease the symptom-related pain, providing relief to patients. There are two basic groups of medication. The first are analgesic and anti-inflammatory drugs, including nonsteroidal anti-inflammatory drugs (NSAIDs) and glucocorticosteroids (GCs). The second are drugs modifying the course of the illness, including biologically active substances. Biologic agents are currently the newest and most advanced form of treatment. They are characterized by a decidedly increased efficacy compared to nonbiologic drugs, as they can more effectively thwart the organism's immunological response, leading to remissions which last several years.

Because there are currently two main methods of pharmacologically treating RA, i.e., using either anti-inflammatory or biologically active drugs, an interesting question arises: does the pharmacotherapy used differentiate patients' pain and levels of anxiety and depression? Additionally, is the level of anxiety and depression determined by a different configuration of psychological variables?

## 2. Methods

### 2.1. Study Participants

85 rheumatoid arthritis patients hospitalized on rheumatoid wards took part in the study. They were aged between 29 and 76 (*M=*48.94;* SD=*14.31). An average of 14.86 years has passed since the initial diagnosis (*SD=*9.31). Women, numbering at 68 (80%), dominated the research group which is consistent with epidemiological data. The participants were treated using biologic agents (57 participants, 67.1%) and standard anti-inflammatory drugs (28 participants, 32.9%). A difference in age has been noted between the treatment groups (*M*_*B*_*=*45.32;* SD*_*B*_*=*13.74;* M*_*NB*_*=*56.32;* SD*_*NB*_*=*12.70;* t*= -3.55;* p*<0.001; Cohen's* d*= 0.83), which was expected, as biological treatment is suggested for younger patients. However, no difference was noted in illness duration (*M*_*B*_*=*14.35;* SD*_*B*_*=*8.28;* M*_*NB*_*=*15.87;* SD*_*NB*_*=*11.24;* t*=0.70;* p*=0.484) nor in gender (women_NB_ 25 (29.21%); men_NB_ 3 (3.53%); women_B_ 43 (50.56%); men_B_ 14 (16.50%); chi^2^=2.250;* p*=0.134).

The study was anonymous and voluntary. It used a questionnaire method. Patients filled out a set of four questionnaires, including the Hospital Anxiety and Depression Scale (HADS) [33], the Ego-Resiliency Scale by Block & Kremen [34]; Polish adaptation: Kaczmarek [30], The Pain Coping Strategies Questionnaire (CSQ) [28], and Visual Analogue Scale [35]. The basic descriptive statistics of the variables measured and the reliability factors are collected in Table 1.

*Hospital Anxiety and Depression Scale* (HADS) [33]: it is made up of two independent subscales, estimating the intensity of anxiety and depression. The scale consists of 14 statements (e.g.,* “I look forward with enjoyment to things*), 7 regarding anxiety and 7 regarding depression. The study participants respond to each statement by choosing one of four possible answers (e.g., 0 as much as I ever did; 1 rather less than I used to; 2 definitely less than I used to; 2 hardly at all).

*The Ego-Resiliency Scale* by Block & Kremen [34]; Polish adaptation: Kaczmarek [30]: the scale is comprised of 14 items measuring the level of resiliency (e.g.,* I like to take different paths to familiar places*). Participants estimate how much each statement applies to them and choose the most appropriate response on a four-point scale, where 1 indicates it does not apply at all and 4 indicates it applies very strongly.

*The Pain Coping Strategies Questionnaire (CSQ)* by Rosenstiel & Keefe; Polish adaptation: Juczyński [34]: the scale consists of 42 statements describing different ways of coping with pain, connected with 7 strategies: diverting attention, reinterpreting pain sensations, catastrophizing, ignoring pain sensations, praying and hoping, coping self-statements, and increasing activity levels. Participants respond using a seven-point scale marking frequency of undertaking the described action, whose extremes are 0 never do and 7 always do that (e.g.,* I don't think about the pain*). Due to the large number of measured coping strategies, in order to conduct detailed analyses the strategies were reduced to three general factors isolated by the method's authors: cognitive coping and suppression, diverting attention and substitute activities, catastrophizing and hoping.

*A Visual Analogue Scale (VAS)* [33] was used to measure the* intensity of pain.* Patients estimated what intensity of pain they felt during the day and at night using two 10-point scales. They marked the appropriate pain level on a horizontal 10-centimeter-long line marking the space between 1 no pain and 10 worst pain imaginable.

## 3. Results

The first step in the analysis was to determine whether RA patients in biological and nonbiological treatments differ in the intensity of pain and levels of anxiety and depression they experience. The choice was motivated by the fact that pain is one of the most troubling physical symptoms of RA, while anxiety and depression are the most disruptive psychologically. To this end, intergroup comparisons were carried out using Student's* t*-test (see Table 2).

The analysis showed that patients undergoing biological treatment exhibited lower pain levels compared to patients treated using standard methods. They were characterized not just by lower overall pain levels, but also lower night and daytime pain. The value of Cohen's* d* suggests that the observed differences are high.

The second stage of analysis was to ascertain the determinants of anxiety and depression for biological and nonbiological treatment groups, separately for each group. This was done in two ways: through calculating simple correlation coefficients for the measured variables and through developing and solving regression equations. Anxiety and depression were placed on the dependent variable side of the regression equation, while the independent variables were daytime pain level, nighttime pain level, ego resiliency, cognitive coping, coping through catastrophizing, and coping through diverting attention.

### 3.1. Determinants of Depression in Patients Undergoing Biological and Nonbiological Treatments

The analysis of depression correlates for biological and nonbiological treatments indicates that there are different factors for either group. Among patients treated using biologic agents, depression levels are related to three variables. They correlate positively with overall (*r*=.27; p=.043) and daytime pain (*r*=.28; p=.036), as well as with ego resiliency (*r*=-.39; p=.002). For patients in the nonbiological treatment group, depression correlates with four variables: positively with daytime (*r*=.39; p=.039) and nighttime pain (*r*=.46; p=.013), but negatively with ego resiliency (*r*=-.44; p=.020) and cognitive coping strategies (*r*=-.49; p=.008) (Table 3).

Two of the variables under consideration turned out to be significant for both treatment groups: overall pain and ego resiliency. Upon comparison, their correlation coefficients appear similar (pain level* z*=.57;* p*=.028; ego resiliency* z*=.52;* p*=.030) (Table 3).

Solving the regression equation for the biological treatment group pointed to the importance of two variables: ego resiliency (*β*=-.41;* p*<.001) and daytime pain levels (*β*=.30;* p*=.014). These two variables combined account for 22% of the variance of depression (*F*=8.72^∗∗^). For patients in the nonbiological treatment group, cognitive (*β*=-.43;* p*=.011) and nighttime pain (*β*=.40;* p*=.018) were significant, accounting for 35% of the variance of depression (*F*=8.24^∗∗^).

### 3.2. Determinants of Anxiety in Patients Undergoing Biological and Nonbiological Treatments

Calculating correlation coefficients made it possible to identify four variables determining the level of anxiety in patients treated with biologic agents. Positive correlations were found for overall pain (*r*=.41; p=.001), as well as its daytime (*r*=.35; p=.008) and nighttime (*r*=.42; p=.001) components. Negative correlations, on the other hand, were found for cognitive coping strategies (*r*=-.33; p=.012) (Table 4).

Among patients treated with nonbiological therapies, anxiety was determined by only one variable: daytime pain (*r*=.41; p=.032). Experiencing daytime pain turned out to be the only anxiety predicate shared by both treatment groups (z=.27; p=.039) (Table 4).

Solving the regression equation for the biological treatment group pointed to the importance of three variables: daytime pain (*β*=.36;* p=*.003), cognitive coping strategies (*β*=-.41;* p*<.001), and coping through catastrophizing (*β*=.26;* p*=.038). This set of three variables accounts for 31% of anxiety variance (*F*=9.53^∗∗^). For patients in the nonbiological treatment group, only daytime pain proved significant (*β*=-.41;* p*=.032), accounting for 13% of anxiety variance (*F*=5.14∗) (Table 5).

## 4. Discussion

The first conclusion that can be drawn from the analyses relates to differences found in pain experienced by patients treated with biologic agents and anti-inflammatory drugs. Analysis results showed that patients receiving biologic agents experience lower levels of pain, both during the day and at night. The noted differences were strong. This shows that using biologically active medication alleviates one of the main symptoms of rheumatoid arthritis, pain, which lets it be assumed that a deferred consequence may be a decrease in depressive symptoms. This assumption is founded on the observation made by Zautra and collaborators [19], who noted that different affective systems were linked to different RA symptoms (pain or impairment). In patients for whom the dominating symptom was pain, an increase in its level leads to an increase in negative affect, without any effect on positive affect. Therefore, using biologic agents should mitigate the vicious cycle mechanism over time. Based on research, the mechanism can be expected to run as follows: pain, appearance of negative affect (primarily depressive symptoms), difficulties in everyday activities including treatment-related activities, which exacerbates the pain. Disrupting this process with an effective form of treatment (biologic agents) which lowers pain levels allows speculation that in the long term, levels of depression will also be lowered and effective function will be increased. The pain experience results indicate which patient group is exposed to greater severity of pain. The results indicate an increased risk group, which should be a premise to prioritise specialist pain management in patients treated with the standard method (anti-inflammatory drugs).

No differences were noted, however, between patients in biological and nonbiological treatment groups regarding their levels of negative affect, anxiety, and depression. This may be related to the study taking place during the treatment process, while changes in mood could be delayed. As such, it has been suggested that future studies on the topic be longitudinal in nature. A different analysis approach may also be considered; namely, the dependency between pain and negative affect could be moderated by the time passed since initiating biological treatment.

The second explored issue dealt with indicators of anxiety and depression in the two treatment groups. A low pain level, personality traits, and strategies of coping with pain were considered potentially beneficial in adapting to chronic illness [11, 36].

It is assumed that pain, one of the most distressing symptoms of RA, has critical influence over the experience of negative emotions, anxiety, and depression. This thesis has been confirmed in previous research [10]. In the current study, the intensity of pain increased the likelihood of a high degree of anxiety and depression both in patients receiving biologic agents and anti-inflammatory drugs. The time at which pain was experienced differentiated the groups, however. For patients treated biologically, pain felt during sleep predicted anxiety, while daytime pain predicted depression. In the anti-inflammatory treatment group, the relationship was inverted; i.e., daytime pain predicted anxiety, while nighttime pain predicted depression.

Ego resiliency, understood as a set of subjective properties governing the ability to flexibly adapt the level of self-control to existing circumstances [29], was taken to be a personality trait pertinent to functioning effectively. The assumption is that it gives sufferers the ability to flexibly choose strategies for coping with pain while taking into account the demands of illness. Previous research has shown that ego resiliency is a key factor in activating positive emotions in difficult situations [37]. The current study aimed to investigate whether ego resiliency has a protective function against negative affect, but the resulting framework of dependencies confirmed this hypothesis only partially. The correlation coefficients obtained confirmed dependencies between ego resiliency and anxiety and depression. However, ego resiliency only demonstrated protective properties against depression in the biological treatment group. Why the variable's positive characteristics only manifested in this group of patients is unknown. The result is made surprising by the fact that previous research on ego resiliency indicated a clear positive role in inducing positive emotional states; it was reasonable to expect resiliency to be a protective factor. Since the present study aimed to describe dependencies between ego resiliency and negative emotional states, it may be suspected that different psychological processes are responsible for arousing negative and positive emotions in the context of a chronic condition. The question of ego resiliency's role in the process of activating negative emotions remains open.

Strategies of coping with pain were the third factor considered as a potential determinant of anxiety and depression. Coping is defined as the individual's efforts to manage arising demands [27]. Previous work has shown that using strategies focused on avoiding and resigning from activity mediate between perceived symptoms of illness and the level of consequent disability and mental disorders [38]. These strategies are linked to a higher risk of developing depression and an increase in the subjective experience of pain, especially in the long-term duration of illness. On the other hand, strategies such as seeking information or support contribute to a higher level of positive affect and better adjustment to illness in RA patients [39]. The present study found that using cognitive coping strategies lowers the likelihood of depression occurring in patients treated with anti-inflammatory drugs and anxiety in patients receiving biologic agents. It was also observed that in the latter group coping through catastrophizing increases anxiety levels. This constellation of dependencies is in line with previous findings [4].

The analysis of relationships between variables showed that the strongest protective function against symptoms is provided ego-resilience and cognitive coping. Probably the role of mental resilience is related to the characteristics of rheumatoid arthritis, which is a very changeable condition. It is not clear which group of strategies for dealing with anxiety and depression symptoms will be effective at a particular stage of the disease, so it is important to be able to adapt to changing conditions, including the flexible use of those strategies. For these reasons, it seems particularly important to develop mental resilience in this group of patients, regardless of the chosen treatment method. Moreover, it seems worthwhile to design and conduct experimental studies in which patients will develop resistance as a result of psychological interventions and check whether its development will help to reduce the symptoms of the disease.

Despite their scientific value, our research has a number of limitations that need to be taken into account when interpreting the results. The limitations of the studies are that they were conducted on a very specific group of hospitalized patients. This implies that the results of the study can only be applied to a group of patients with acute symptoms of rheumatoid arthritis. In future studies, analyses should also be carried out on other groups of patients, i.e., outpatient clinics. In addition, patients were treated as a homogeneous group in regard to the severity of the disease. In future studies, it would be worth taking into account the severity of the disease estimated, e.g., by means of the DAS—Disease Activity Score—a scale for assessing the level of activity of symptoms of rheumatoid arthritis. As well as check if the disease activity interacts with the other variables studied.

## Figures and Tables

**Table 1:** Comparison of pain, depression, and anxiety among patients in biological and nonbiological treatment: *t*-Test of differences for independent samples.

**Variable**	**Treatment type**		***t-*Test statistics**			
	**biological treatment (*n*=57)**	**nonbiological treatment (*n*=28)**	***t***	***df***	***p***	***d***
	***M* (*SD*)**	***M* (*SD*)**				

*Pain intensity*	8.3 (4.94)	12.12 (4.85)	3.36	83	.001	.78
Daytime pain	4.71 (2.49)	6.80 (2.18)	3.78	83	<.001	.90
Night pain	3.60 (2.76)	5.32 (2.99)	2.62	83	.010	.60

*Anxiety*	8.26 (3.05)	9.21 (3.82)	1.24	83	.218	-
*Depression*	5.05 (3.21)	6.28 (3.42)	1.63	83	.107	-

**Table 2:** Descriptive statistics, reliability coefficient Cronbach's alpha.

	**Range**	***M***	***SD***	***α***

1. Pain intensity	0-20	9.57	5.20	.78^∗∗^
a. Daytime pain	0-10	5.40	2.58	-
b. Night pain	0-10	4.17	2.93	-
2. Ego-Resiliency	24-55	41.67	7.03	.84
3. Cognitive coping	2-99	46.93	20.78	.91
4. Distraction coping	0-63	32.76	14.20	.88
5. Catastrophizing coping	0-64	31.36	13.67	.82
6. Anxiety	1-19	8.58	3.33	.76
7. Depression	0-13	5.46	3.31	.82

^*∗∗*^the size of the correlation coefficient is reported because the intensity of pain was determined on the basis of two items.

**Table 3:** Correlation matrix. Correlation between pain intensity, mental flexibility, coping, anxiety, and depression.

	**1**	**1a**	**1b**	**2**	**3**	**4**	**5**	**6**	**7**

	*Biological treatment*								

1. Pain intensity		.93^∗∗^ (p<.001)	.95^∗∗^ (p<.001)	.04 (p=.777)	.07 (p=.584)	.17 (p=.199)	.29^∗^ (p=.028)	.41^∗∗^ (p=.001)	.27^∗^ (p=.043)
a. Daytime pain	.92^∗∗^ (p<.001)		.77^∗∗^ (p<.001)	.05 (p=.698)	.12 (p=.389)	.13 (p=.323)	.25 (p=.063)	.35^∗∗^ (p=.008)	.28^∗^ (p=.036)
b. Night pain	.96^∗∗^ (p<.001)	.76^∗∗^ (p<.001)		.02 (p=.876)	.03 (p=.838)	.19 (p=.160)	.30^∗^ (p=.024)	.42^∗∗^ (p=.001)	.23 (p=.087)
2. Ego-Resiliency	-.01 (p=.957)	.01 (p=.977)	-.02 (p=.912)		.24 (p=.070)	.23 (p=.081)	-.08 (p=.555)	-.24 (p=.075)	-.39^∗∗^ (p=.002)
3. Cognitive coping	-.16 (p=.420)	-.14 (p=.472)	-.15 (p=.433)	.40^∗^ (p=.038)		.76^∗∗^ (p<.001)	.28^∗^ (p=.035)	-.33^∗^ (p=.012)	-.18 (p=.175)
4. Distraction coping	-.01 (p=.957)	.01 (p=.977)	-.02 (p=.914)	.59^∗∗^ (p=.001)	.55^∗∗^ (p=.003)		.28^∗^ (p=.038)	-.09 (p=.505)	-.18 (p=.179)
5. Catastrophizing coping	.34 (p=.076)	.41^∗^ (p=.029)	.25 (p=.195)	.14 (p=.480)	.18 (p=.372)	.25 (p=.205)		.25 (p=.062)	.26^∗^ (p=.048)
6. Anxiety	.36 (p=.091)	.41^∗^ (p=.032)	.23 (p=.235)	-.18 (p=.354)	-.11 (p=.592)	-.04 (p=.840)	.37 (p=.054)		.39^∗∗^ (p=.003)
7. Depression	.39^∗^ (p=.039)	.24 (p=.227)	.46^∗^ (p=.013)	-.44^∗^ (p=.020)	-.49^∗∗^ (p=.008)	-.17 (p=.376)	-.11 (p=.581)	.20 (p=.303)	

	*Nonbiological treatment*								

^*∗*^ *p* < 0,05, ^∗∗^*p*< 0,01, correlation coefficients for patients in biological treatment are reported in upper right side of the table; on the contrary, correlation for patients in nonbiological treatment is showed in lower left side of the table.

**Table 4:** Anxiety and depression predictors among patients in nonbiological treatment, stepwise regression analysis results.

**Anxiety**				**Depression**			
**Predictor**	*β*	*t*	*p*	**Predictor**	*β*	*t*	*p*

Daytime pain	.41	2.27	.032	Cognitive coping	-.43	-2.,75	.011
				Night pain	.40	2.53	.018
*R* ^*2*^ = .13, *F *= 5.14^∗^				*R* ^*2*^ = .35, *F *= 8.24^∗∗^			

**Table 5:** Anxiety and depression predictors among patients in biological treatment, stepwise regression analysis results.

**Anxiety**				**Depression**			
**Predictor**	*β*	*t*	*p*	**Predictor**	*β*	*t*	*p*

Night pain	.36	3.07	.003	Ego-Resiliency	-.41	-3.45	.001
Cognitive coping	-.41	-3.56	.001	Daytime pain	.30	2.53	.014
Catastrophizing coping	.26	2.12	.038				
*R* ^*2*^ = .31, *F *= 9.53^∗∗^				*R* ^*2*^ = .22, *F *= 8.72^∗∗^			

## Data Availability

The data used to support the findings of this study are available from the corresponding author upon request.
